# “They’ll Talk About Everything Else… But Suicidal Ideation”: Clinician Experiences Addressing Non-Disclosure of Suicidal Ideation Among Military-Affiliated Clients

**DOI:** 10.1007/s11606-025-09570-y

**Published:** 2025-05-28

**Authors:** Melissa A. Litschi, Steven L. Lancaster, David J. Linkh, Megan Lafferty

**Affiliations:** 1 Cohen Veterans Network, Institute for Quality, Stamford, CT USA; 2 Center to Improve Veteran Involvement in Care (CIVIC), VA Portland Health Care System, Portland, OR USA

**Keywords:** suicidal ideation, suicide assessment, disclosure, Veterans, mental health clinicians, qualitative

## Abstract

**Background:**

More than half of people experiencing suicidal thoughts and behaviors may never disclose their experiences to another person. Veterans are more likely to die by suicide than their civilian counterparts and report barriers to disclosure of suicidal thoughts and behaviors during screenings. While studies of veteran and service member perspectives offer recommendations to facilitate disclosure, little is known about clinician perspectives and strategies.

**Objective:**

Describe clinician perspectives on non-disclosure among military-affiliated clients and strategies to address potential non-disclosure in this at-risk population.

**Design:**

Qualitative analysis of transcript summaries from semi-structured interviews.

**Participants:**

Seventeen clinicians serving military and veteran clients participated. Professional backgrounds and credentials were diverse, with 71% having 5 or more years of clinical experience. Roughly half of the participants treated clients with suicidal ideation three or more times per week.

**Approach:**

Interviews focused on clinicians’ approaches and decision-making processes during suicide risk stratification and treatment planning, including barriers and facilitators. This paper focuses on identified challenges of non-disclosure. Transcripts were analyzed using rapid qualitative analysis.

**Key Results:**

Clinicians described their experiences with non-disclosure of suicidal ideation among military-affiliated clients, including perspectives on disclosure barriers and communication strategies used to facilitate disclosure. When discussing the challenge of non-disclosure clinicians reported (1) experiencing guardedness and non-disclosure among their clients, and (2) perceiving stigma and fear of negative consequences as disclosure barriers. Based on these experiences, clinicians modified their approaches to suicide risk assessment to facilitate disclosure by (1) normalizing suicidal thoughts and behaviors as safe topics, (2) educating clients to address fears, (3) collaborating with clients to promote acceptance of safe firearm storage, and (4) deliberately using standardized measures to overcome disclosure challenges.

**Conclusions:**

Proactively implementing communication strategies that address perceived barriers to disclosure of suicidal thoughts and behaviors among military-affiliated psychotherapy clients may facilitate disclosure.

## INTRODUCTION

Suicide risk assessment is a well-established element of suicide prevention and universal screening using standardized assessment tools is considered a best practice across a variety of health care settings from behavioral health to primary care and emergency departments.^1,2^ However, providers have identified important challenges to the suicide risk assessment process,^3–5^ including concerns over accurate disclosure of suicidality by mental health patients.^4^ Veteran suicide rates are 57.3% higher than their civilian counterparts,^6^ with the majority of suicides among veterans (73%)^7^ and active duty service members (65%)^8^ involving firearms. Firearm ownership is common among military-affiliated individuals^9,10^ (veterans and military service members), who often store at least one household firearm loaded and unlocked, increasing risk.^11^ Additional risk factors among military-affiliated individuals include post-traumatic stress disorder (PTSD), military sexual trauma, and traumatic brain injury.^12,13^ Within the Veterans Health Administration (VHA), universal screening has been linked to improved engagement in mental health care for veterans.^14^ Despite widespread acceptance of universal suicide risk screening, military-affiliated individuals report barriers that may prevent disclosure during screenings.^15–17^

Previous studies have shown that patients frequently hesitate or do not disclose suicidal thoughts and behaviors (STB) during suicide screening assessments with medical and mental health providers, with a recent review of nearly 100 studies indicating that over 50% of people do not disclose STB.^18^ Blanchard and Farber^19^ found that 31% of study participants reported lying to a therapist about suicidal ideation in the past and 10% reported lying about a previous suicide attempt, while Morrison and Downey^20^ found that 14% of participating college students reported denying suicidal ideation on an intake questionnaire but later disclosed suicidality to a therapist. More recently, Sagmo Høyen and colleagues^21^ reported 51.5% of psychiatric patients withheld information about suicidal thoughts during intake. Additionally, in a sample of VHA patients, 72% of individuals who died by suicide during the 1-year study period did not report suicidal ideation on the Patient Health Questionnaire (PHQ-9),^22^ although it is unknown whether these responses represent non-disclosure or a change in symptoms.

In studies of patient perspectives, reasons for non-disclosure of suicidality included perceived stigma surrounding the topic and fears of negative consequences across a range of populations, including college students,^23^ primary care patients,^24^ psychotherapy clients,^25,26^ and military members and veterans.^15–17^ Self-stigma, internalized negative stereotypes of STB, and anticipated stigma, fear of judgement from others, were both associated with a reduced likelihood to disclose STB among veterans but not among civilians.^27^ Fear of hospitalization was reported as a leading motivator for concealing even mild suicidal thoughts by psychotherapy patients,^25,26^ while primary care patients reported making active choices about disclosure after considering their desire for and likelihood of receiving support compared to their fears of negative consequences.^24^ For service members, treatment seeking and disclosure may be affected by stigma, a desire to avoid appearing burdensome, concerns over the impact on social relations, and legitimate fears over occupational impacts such as career advancement or negative views by leadership.^15,28^ In a study of veterans, participants reported denying STB on prior screens due to shame around the topic, beliefs that suicidal thoughts are private, and fears related to hospitalization.^17^ Additionally, these veterans reported feeling that screenings were just checking a box, did not allow patients to share the complexity of their experiences, and did not influence their care.

Fewer studies have been conducted of provider perspectives of patients’ motivations for non-disclosure, which may impact provider approaches to risk assessment. In one study, emergency department providers reported beliefs that honesty during risk assessment was hindered by lack of privacy from family, defensiveness in response to monitoring by police or security, and desire to avoid psychiatric hospitalization.^5^ In another, mental health clinicians reported beliefs that hesitation over disclosure was due to fears of negative consequences including scaring the provider, uncertainty of outcomes, and loss of control, as well as cultural stigma and taboos around discussing suicidality.^4^ Clinician insights gained through clinical experience provide a unique window into the phenomena relating to non-disclosure that may help to enrich non-disclosure literature based on a limited number of firsthand studies of patient perspectives. Additionally, understanding perspectives that inform clinical responses may help to elucidate strategies used by clinicians faced with uncertainty and the potential for non-disclosure during risk assessment.

While existing research makes recommendations for addressing non-disclosure during suicide risk assessments,^16,25,29^ no studies have examined the approaches providers employ to encourage disclosure based on their perceptions about client motivations to deny STB. This paper examines clinician experiences with and responses to non-disclosure of STB among military-affiliated clients in a mild to moderate acuity outpatient mental health setting. The purpose of this study was to better understand the complex process of suicide risk assessment in clinical practice. Specific aims were twofold:

1. To explore clinician perceptions and beliefs about client motivations for non-disclosure of STBs
2. To describe the strategies employed by clinicians to facilitate disclosure of STBs

## METHODS

We conducted semi-structured interviews as a part of a mixed-methods, vignette-based study on suicide risk stratification in an outpatient mental health setting.^30^ These interviews had two aims: (1) to understand participants’ individual perspectives and approaches to risk assessment in clinical settings and (2) to elicit additional details about factors that influenced risk and treatment determinations in the vignette-based assessment exercise previously completed by participants. Interviews were conducted virtually between May and July 2023.

### Study Setting

Research was conducted within Cohen Veterans Network (CVN), which currently operates 22 mental health clinics serving military service members, veterans, and their family members across the USA. CVN has implemented a robust clinical suicide risk management ecosystem that includes training, evidenced-based universal screening, and measurement-based care procedures. All CVN clinicians are required to complete a suicide prevention foundations training within 6 months of onboarding at a Cohen Clinic, with additional evidence-based suicide intervention trainings and regularly occurring consultations with subject matter experts available. Universal screening for suicide risk occurs at intake using the Columbia Suicide Severity Rating Scale (C-SSRS) Screener – Recent version which involves a two-item screener (questions 1 and 2) completed by the front desk staff. A positive screen on this measure prompts an immediate warm handoff to a clinician to complete the remaining questions of the C-SSRS Screener to assess for immediate safety concerns and develop safety plans as appropriate. Clients are again screened for risk using the C-SSRS Lifetime/Recent version during an initial biopsychosocial interview. This interview is a comprehensive assessment of the client’s history (e.g., education, employment, mental health history, medications) as well as their current symptoms, presenting problem, and functioning. This screening and assessment process is consistent across adult client types, for example veterans, military service members, and family members are assessed similarly. Active duty clients receiving care through a Tricare referral are required to sign a release of information allowing communication with the referring military treatment facility. Clients assessed at elevated risk based on a robust assessment including C-SSRS scores, history, and present functioning are placed on an elevated risk pathway, in which they collaboratively develop and maintain a safety plan with their assigned therapist, are offered suicide-specific interventions, and are monitored through suicide specific screeners and standardized symptom measures at each session.

Measurement-based care, a protocol that involves systematic collection of patient-reported outcome measures, collaborative review with a clinician at each session, and continuous reassessment of the treatment plan in accordance with the results,^31^ is a cornerstone of CVN’s treatment model and incorporates several different standardized and validated measures, depending on the unique needs and symptoms of the client. Clients on CVN’s elevated risk pathway complete the PHQ-9 and C-SSRS (Since Last Visit version) during weekly sessions. Clients who are not assessed at elevated risk complete diagnostic specific assessments weekly. These assessments are intended to complement clinical judgment in the identification and monitoring of risk and in treatment planning.

### Study Participants

Mental health clinicians employed at Cohen Clinics were eligible for this study. Cohen clinicians are employed by partner organizations, like a franchise model. Prospective participants were recruited for a study of clinical decision-making related to suicide risk stratification via email.^30^ Clinicians who completed the vignette assessment component (*n* = 42) were invited to complete a follow-up interview, with 22 clinicians volunteering. Participants were not compensated for their participation in the study.

### Data Collection and Analysis

We conducted 60-min semi-structured interviews covering topics relating to clinician’s approaches and decision-making processes when stratifying suicide risk and making treatment determinations. As noted above, these interviews were conducted as a follow-up to a project in which participating clinicians had been asked to evaluate risk in hypothetical client vignettes. However, the interview portion of the study focused primarily on participants’ general experiences with and approaches to suicide risk assessment and management. Clinicians were specifically asked to discuss their approaches to suicide risk assessment, factors which facilitated or challenged this process, their prioritization of risk and protective factors, the impact of risk assessment on treatment planning, and experiences conducting risk assessments with military affiliated clients. Informed consent was obtained prior to beginning the interview after participants were given the opportunity to ask questions about the project and informed consent documents. The study protocol, including the recruitment and informed consent process, was deemed exempt by the WCG Institutional Review Board (Protocol Number: CVN-2). Interviews were conducted by the first author, who has expertise in qualitative interviewing and analysis from an anthropological perspective. Zoom was used to conduct, record, and transcribe all interviews. Interview transcripts were corrected and verified by the first author.

Interview transcripts were analyzed using Rapid Qualitative Analysis, a structured qualitative approach^32^ with results comparable to more time-intensive thematic analysis.^33,34^ This method has demonstrated utility in health services research where rigorous qualitative insights are often needed to inform clinical policy and training.^35–38^ Data analysis followed an iterative process involving discussions with the team members to develop transcript summary templates and generate themes. Following the transcription and review of the first six interviews, the lead author developed a summary template with domains (key topics) drawn from the interview questions. Two qualitative analysts (MAL and ML) then summarized three selected interviews, made notes on summary template organization and usability, and collaboratively revised the template. The summary template was finalized with input from the broader mixed-methods project team, who commented on domain formulation based on their clinical and research expertise. The two qualitative analysts then revised summaries of the initial three interview transcripts, meeting to confirm reliability of summaries and discuss inconsistencies. The remaining transcripts were divided between the two qualitative analysts and summarized. Analysis of the transcript summaries was completed by the first author, and identified themes were discussed between the two qualitative analysts and presented to the broader research team on a regular basis. The first author then met with the broader research team who reviewed transcript summaries from a clinical perspective with to determine the validity of preliminary themes and actionable findings relevant to clinical policy and practice. For the data presented in this paper, the first author coded transcript summaries related to themes of hesitancy to disclose STB and clinical strategies to encourage disclosure. Interpretations of the data were reviewed and discussed by the authors to ensure consensus was reached with all authors in agreement regarding themes.

## RESULTS

Seventeen Cohen clinicians completed semi-structured interviews. Participant characteristics, including demographics and clinical experience, are summarized in the participant characteristics table (Table 1). Participant expertise in suicide assessment and intervention was high, with 71% of participants having 5 or more years of clinical experience and 47% treating clients with suicidal ideation 3 or more times per week. All participants completed CVN’s suicide prevention foundations training prior to participating in this study.

**Table 1:** Participant Characteristics

Characteristic	*N* = 17*
Credentials	
Licensed clinical social worker (LCSW)	8 (47%)
Licensed master social worker (LMSW)	1 (5.9%)
Licensed marriage and family therapist (LMFT)	2 (12%)
Licensed professional counselor (LPC)	5 (29%)
Psychologist	1 (5.9%)
Years’ experience	
1–2 years	1 (5.9%)
2–5 years	4 (24%)
5–10 years	9 (53%)
> 10 years	3 (18%)
Gender	
Woman	16 (94%)
Frequency treating clients with SI	
1–2 times per every 6 months	1 (5.9%)
1–2 times per month	5 (29%)
1–2 times per week	3 (18%)
3 + times per week	8 (47%)
Frequency placing high risk flag	
1–2 times per every 6 months	2 (12%)
1–2 times per month	8 (47%)
1–2 times per week	4 (24%)
1–2 times per year	2 (12%)
3 + times per week	1 (5.9%)
Frequency developing safety plan	
1–2 times per year	1 (5.9%)
1–2 times per month	8 (47%)
1–2 times per week	5 (29%)
3 + times per week	3 (18%)

^*^n (%)

Findings are organized around participating clinicians’ perspectives of barriers to disclosure of STB during suicide risk assessment and communication strategies to facilitate disclosure. Clinicians identified non-disclosure as a challenge in the risk assessment process among military-affiliated clients based on (1) experiencing guardedness and non-disclosure among their clients and (2) their perceptions of client barriers to disclosure. Communication strategies they used to address non-disclosure encompass four approaches to create a safe atmosphere for sharing suicidal thoughts and behaviors, including (1) normalizing STB as a safe topic, (2) educating clients to address fears of negative consequences, (3) collaborating with clients to promote acceptance of safe firearm storage, and (4) deliberately using standardized measures to overcome disclosure challenges.

### Challenges and Barriers to Disclosure of STB When Working with Military-Affiliated Clients

Difficulties with risk assessment and treatment — Honesty, some people aren’t honest with you for whatever reason. That can be difficult.... What else is difficult? I don’t know. That’s the only thing I can think of. [LCSW, 5–10 years’ experience]

#### Experiences with Guardedness and Non-Disclosure Among Military-Affiliated Clients

During the suicide risk assessment process with military and veteran clients, many participating clinicians faced challenges related to guardedness and reticence around reporting suicidal ideation, often leading to delayed- or non-disclosure. Participating clinicians felt that many veteran and military clients entered mental health care with a “we can’t talk about these things” approach or by “[talking] about everything else that’s bothering them but suicidal ideation.”

Examples of delayed and non-disclosure varied substantially in both timing and resolution. Participating clinicians reported that some military-affiliated clients would deny suicidal ideation on screeners during their intake process but later open up about their suicidal thoughts after several therapy sessions, once rapport was better established.

I have had some situations where I've been working with somebody 3, 4, 5 sessions, and they have never brought it up. They deny it on the screens, and they don't bring it up. And then we're in some space, and for whatever reason they feel safe enough at that moment to say, “Well, by the way, I have been doing this.” [LCSW, 2–5 years’ experience]

In other cases, participating clinicians reported that military-affiliated clients would often respond “no” to typical screener questions about suicidal thoughts, but then answer “yes” when asked directly about engaging in suicidal behaviors or if they had thought about ways they might kill themselves. For example, one clinician estimated that roughly one in ten military and veteran clients deny STB on all screener questions but endorse question 6 of the C-SSRS “have you done anything, started to do anything, or prepared to do anything to end your life?^39^”.

#### Perceived Disclosure Barriers Include Stigma and Fears of Negative Consequences

Based on clinical experiences, participating clinicians attributed motivations for guardedness or non-disclosure of suicidal thoughts to ongoing cultural stigma surrounding suicide in the military community and fears of the consequences of reporting suicidal ideation.

...a couple of weeks down the road… when you’re addressing again… that’s when you learn like they’ve been having [suicidal thoughts] for a while. They were just afraid to say that. [LMSW, 1–2 years’ experience]

Participating clinicians concluded that many clients struggled with stigma surrounding their suicidal ideation, including worrying about being judged by other servicemembers or veterans, feeling like their ideations were a weakness, or struggling to overcome the mentality that suicide was just not something to discuss—“…what we find is that it's that stigma piece. It's the belief that everybody is judging me.” Clinicians expressed concern that this perceived stigma continued to impact clients despite work to normalize seeking help for suicide within the military, while others noted that younger veterans and service members appeared more willing to discuss these experiences than older veterans.

And supposedly they’re trying to normalize it, you know, psychology and all that in the military. But it doesn’t seem like they really are. And so there’s still a big stigma around that. And so a lot of people are suffering in silence. [LMFT, 10+ years’ experience]

…not necessarily the younger population, the younger soldiers, but the older soldiers or like the ones who’ve been in for a long time, maybe came through at a time when you didn't talk about it right?... It’s more that population, because there is just such a stigma against even mental health treatment. [LPC, 2–5 years’ experience]

Participating clinicians also described fears their clients expressed over the consequences of endorsing suicidal ideation. Clinicians cited fears about hospitalization and loss of independence as important reasons that may drive guardedness and hesitancy to disclose suicidal ideation among service members and veterans. Clinicians perceived these fears to be driven both by personal past experiences and by prevailing sub-cultural narratives or beliefs encountered by clients.

I had a number of service members who have talked about suicide at the VA or on post. The next thing you know, they’re being escorted [to inpatient psychiatric care].… that’s not everyone’s experience of course, but that is an experience that more people have than I would like. [LCSW, 5–10 years’ experience]

Similarly, participating clinicians perceived that service members and veterans are particularly uneasy about the possibility of losing access to firearms, which may impact both disclosure of STB as well as their receptivity to lethal means counseling. Some clinicians described strong feelings past clients have expressed surrounding their firearms. These clinicians expressed that firearms play an important role in promoting a feeling of safety among service members and veterans with PTSD. Additionally, clinicians cited political beliefs related to gun rights as perceived factors contributing to concerns relating to loss of access. In one case, a participant reported concerns over loss of firearm access was related to fears of others finding out about clients’ suicidal ideations.

Like a lot of times [veterans] don’t want to say anything because you’re gonna come in and take [their] guns away… and a lot of them feel very political, you know, that's their rights. And a lot of them, if they have PTSD, it’s a safety feature for them to have [firearms] in a home… they feel more confident having a firearm in the home. [LCSW, 5–10 years’ experience]

For military clients, participating clinicians described perceived worries about how disclosing suicidal ideation will impact their careers and mission-readiness, including concerns over the privacy of their medical records. Some speculated that, for service members, feared career impacts are ultimately linked to fears about the impact on their self-image and sense of purpose.

Soldiers struggle also with support because they feel once they start going through some major issues that their command and everyone else pretends to care until they’ve determined they’re going to be kicked out of the military, and they’ve, they’ve lost their purpose. [LPC, 5–10 years’ experience]

I feel like it’s been so pressed on them through their military career that if you are linked to mental health, you are going to lose your job…. So sometimes I think you have to… be aware of it, and be able to work with that, to get to where you need to go. [LCSW, 5–10 years’ experience]

### Communication Strategies to Address Disclosure Challenges

Participating clinicians discussed several strategies they use, in conjunction with suicide risk assessment, to address non-disclosure. Often these adaptations are applied to all clients but appear driven by challenges encountered working with military-connected clients. These adaptations center around modifications in their communication styles, adaptations to client education on the limits of confidentiality, collaborative approaches to lethal means counseling, and deliberate use of standardized measures as a communication tool.

#### Normalizing STB as a Safe Topic

Many participating clinicians remarked that it can be difficult for people to talk about suicide, both for clients experiencing STB and for themselves as clinicians. They described their efforts to be confident discussing the topic so clients can be comfortable sharing their experiences. Clinicians often reported introducing the topic of suicide early and using direct and explicit language to avoid euphemisms that may reinforce the idea that the topic of suicide was taboo. Clinicians believe this helps normalize suicide as a safe topic of conversation. These clinicians hope that by demonstrating their own comfort and experience discussing and treating suicidal ideation that they can help their clients feel more at ease opening up and sharing their own experiences and concerns.

I think that because of the stigma and the taboo around it, it makes it really hard for people to talk about, and when I first started working in mental health it was really hard for me to talk about. So I get that. But now, just making it a regular part of conversation. [LMSW, 1–2 years’ experience]

My belief is that, it starts to kind of normalize it, I use the words right away, and so they’re not taboo. I talk about killing yourself and suicide, and not just harming yourself. [LCSW, 5–10 years’ experience]

It happens, people have suicidal ideation. And when they can see that you’re comfortable talking about it, you’re not afraid to approach it, to address it. You’re not afraid of what they’re gonna say in response to it. Then it becomes, you know, easier for them. [LMSW, 1–2 years’ experience]

#### Educating Clients to Address Fears of Negative Consequences

Given the perceived hesitancy to disclose suicidal ideation due to fears about negative consequences, many participating clinicians also described adaptations they have made to their discussions around limits of confidentiality and the purpose of risk assessment. One of the points clinicians find important to clarify is the threshold for recommending voluntary or involuntary hospitalization. These clinicians highlight the importance of client safety and the ways in which suicidal ideation and behaviors can be addressed through outpatient therapy.

I tell folks like, just because you’re having suicidal thoughts doesn’t necessarily mean that... I’m going to have to hurry up and send you to the hospital. We can work together to figure out what’s pushing you towards the suicidal thoughts. [LCSW, 5–10 years’ experience]

According to participating clinicians, discussions around limits of confidentiality may also include acknowledging experiences with or fears about sudden, involuntary hospitalization on base or at the VHA, describing differences between on and off base procedures, and educating clients about federal and state laws that impact clinician responsibilities to report. Clinicians also report finding value in assuring clients that if hospitalization ever became a possible recommendation, there would not be any surprises, and that this decision would be discussed collaboratively with the client. Participating clinicians believe these conversations help to build rapport and trust between the client and the clinician. To maximize the impact of these adaptations, several participants emphasized that these conversations should be initiated early during the first meeting and reiterated frequently throughout the course of care.

I talk about privacy is paramount, but there are certain situations where safety trump’s privacy. One of those is imminent risk towards killing yourself or harming yourself. And the word there is imminent…. I try to really illustrate that there’s a lot of room for this conversation to exist in a way that doesn’t have to sound every alarm bell and take independence right away. [LCSW, 5–10 years’ experience]

I think it strengthens the relationship from the very beginning. They’re sharing some really difficult things. But then also it helps give me that understanding. And then, you know, just talking that through with, “Hey, this isn’t information that I’m going to use against you. It’s information for us to be able to improve your care.” [LPC, 2–5 years’ experience]

#### Collaboration to Promote Acceptance of Safe Firearm Storage

Similarly, participating clinicians addressed possible concerns over loss of firearms with clear and direct communication that focused on safety and the goals of safe firearm storage. One clinician spoke extensively on their approach to lethal means counseling with veterans. In conversations with clients, this clinician explains that the goal is to increase safety, not take away a client’s guns. They collaborate with clients to find solutions that both address safety and account for client concerns about firearm access, with many clients offering creative solutions that meet their needs. Participating clinicians found that most clients were open to some form of safety measures when they were a collaborative participant in this element of safety planning.

I think when you explain the rationale and make sure you lead with “I am not trying to take away your rights and not trying to take away your guns. I am just trying to help you through this specific period of time that you're not that you're not doing well”…. And I, knock on wood, have not had someone that said “hell no” to every suggestion I had. They usually walked away implementing something…. We figure something out together. [LCSW, 5–10 years’ experience]

#### Deliberate Use of Standardized Measures to Overcome Disclosure Challenges

Participating clinicians endorsed clinic and network level policies around measurement-based care as both a mechanism to facilitate suicide risk assessment and, specifically, as an aid to overcome challenges regarding honest and timely disclosure of suicidal ideation and behaviors. They emphasized that standardized measures alone could not provide a complete picture of a client’s symptoms and experiences, rather, these measures are tools that facilitate clinical interviewing and build therapeutic alliance. Connection or partnership with the client remains a critical aspect in risk identification and mitigation.

Standardized assessment tools, such as the C-SSRS and PHQ-9, were described as “conversation starters,” introducing and normalizing the topic of suicide. Clinician participants report that these measures provide a direct avenue to introduce the topic of suicide and help to elicit information a client may not offer without prompting. These standard measures provide a foundation for clinical interviewing where clinicians can more thoroughly assess a client’s risk. For example, positive responses to items from standardized measures used in initial assessments highlight areas where the clinician needs to elicit additional details to build an understanding of the full context of a client’s experiences. Even negative responses offer an opportunity to follow-up if the clinician feels a client’s affect does not align with their verbal response. Additionally, weekly, or periodic measures for clients who do not screen at high risk, require clinicians to regularly revisit the topic of suicide and provide additional opportunities for clients to disclose suicidal ideation or behaviors after building a relationship with the clinician.

I 100% believe in measurement-based care and doing the measures every session. Yeah, because I have noticed that people don’t, they don’t share as much as was revealed on there and then that kind of starts a conversation. [LMFT, 10+ years’ experience]

Participating clinicians discussed how the differently phrased questions on these assessments often helped lead to discussions that reveal suicidal thoughts or behaviors that were not disclosed on initial screeners. In some cases, they attributed this change in disclosure to variations in phrasing or conceptualization of suicidal ideation that resonated with different clients, helping them to recognize certain thoughts or actions as suicidal ideation. Some clinicians even used answers on screeners for anxiety (GAD-7) or post-traumatic stress (PCL-5) to elicit information about risk factors that are commonly associated with suicidal ideation, aiding clinicians in their clinical interviews and risk assessments.

[They] kind of give me a greater idea of potential risk that I want to take into consideration. And especially with our population…coming in with this... “We can’t talk about these things”. That’s an, that’s an area that I have found very helpful. [LMFT, 5–10 years’ experience]

In other cases, including for military-connected clients, some participating clinicians thought that clients became habituated to the practice of denying suicidal ideation in traditional screening questions and so more novel or behavior-focused questions were able to elicit more information. Based on responses to these measures, clinicians were able to follow up with additional questions to draw out more details on the experiences and thoughts the client may be struggling with.

I’m a pretty big research nerd myself, so I’m like very into reading the assessment as a written, and one of the things I say is to continue to read the behavior section [of the C-SSRS], even if they said “No” to everything. And with the military population the amount of times I’ve caught people, for lack of a better phrase, in the second section… They’re always answering the first 2, if not the first 6. And so some of those newer questions on the back end of the C-SSRS really give me more information. [LCSW, 5–10 years’ experience]

Not only is the use of information gathered through standardized measures important, but delivery of the measures also matters as well. Some participant clinicians, particularly those in supervisory or training roles, reported observing a tendency for clinicians to disconnect when administering standardized assessments, harming therapeutic alliance. Ultimately, participating clinicians viewed standardized measures as a tool to support more collaborative conversations about the client’s experiences and treatment goals.

No tool or no risk assessment can help you if you’re not going to connect with the human in front of you. [LCSW, 5–10 years’ experience]

## DISCUSSION

This qualitative study aimed to improve understandings of clinicians’ perspectives on non-disclosure of suicidality, its impacts on risk assessment, and strategies used to address these challenges. Participating clinicians believe that the potential for non-disclosure of STB was a major challenge in the risk assessment process for military and veteran clients, with many experiencing some form of delayed disclosure among their own clients. They attribute guardedness around STB to persisting stigma in the military and veteran communities, including fears of being weak (self-stigma) and fears of being judged by other service members and veterans (anticipated stigma), as well as to fears of negative outcomes, including loss of independence or unwanted hospitalization, loss of firearm access, and adverse career impacts.

Participating clinician experiences with delayed disclosure align with prior studies both among the general clinical population^18–20^ and veteran and active duty study participants,^15–17,40^ including desires to avoid stigma, negative public perception, and unwanted negative consequences.^15–17,23,25,27^ Among veterans specifically, study participants’ perceptions of motives for non-disclosure align with those most cited in studies of veteran perspectives, including shame and perceptions of weakness, beliefs that suicidal ideation is a private or personal matter, fears about involuntary hospitalization or treatments, and dislike of cursory screenings as factors in their decisions to not disclose suicidal ideation.^16,17^ However, studies of veterans’ perceptions demonstrate that disclosure is context dependent, with participants more likely to disclose when they have a relationship with a trusted provider.^16,17^ Further, a study of National Guard personnel found that participants may be more likely to disclose STB on screeners that they know are not linked to their military record,^40^ echoing concerns reported by clinicians in the present study.

Based on experiences with guardedness and delayed disclosure, participants in the present study implemented four key communication strategies to address non-disclosure among military-affiliated clients. These include normalizing discussions of STB with direct language, addressing perceived motivations for non-disclosure through education around treatment goals and limits of confidentiality, addressing safe firearm storage collaboratively and reassuring clients about the purpose, and deliberately utilizing standardized measures as communication and rapport-building tools. These approaches align with patient recommendations to create safe reporting spaces by explaining triggers for hospitalization and inclusion in decisions to escalate care.^25^ Additionally, they may promote contexts where military-affiliated clients feel safe disclosing STB by explaining the purpose of suicide risk assessment and limits of confidentiality, as well as focusing on building a therapeutic relationship.^16,17,40^ Strong therapeutic relationships between patients and providers have been found to improve disclosure. Farber and Hall^41^ found that greater therapeutic alliance was positively correlated with overall disclosure of suicidality, while Sagmo Høyen and colleagues^21^ found that therapists’ emotional responses to patients correlated with non-disclosure among psychiatric inpatients. These strategies also align with initiatives such as those led by True and colleagues,^42^ which involve partnering with gun stores to provide firearm storage options and distributing gun locks to veterans at risk of suicide. Such efforts offer additional strategies for clinicians—both in mental health and primary care settings—to facilitate open discussions around firearm safety while maintaining trust and respecting clients’ autonomy.

While participating clinicians found significant benefits in the use of measurement-based care during suicide risk assessment and management, Ganzini and colleagues^17^ reported impersonal and perfunctory standardized measures contributed to veteran decisions to not disclose suicidal ideations. Clinicians in the present study also cautioned about the loss of connection when administering standardized assessment measures and inability of measures to tell the full story of a client’s experience. Rather, they viewed these measures as important “conversation starters” leading to collaborative conversations with clients. This complementary approach was seen as essential to the risk assessment process. During conversations and clinical interviews, clinicians could follow-up on any disclosed symptoms or experiences to build a more complete understanding of the client’s experience with suicidality, including history, severity, frequency, nuances, and existing coping skills. Collaborative conversations were also critical to the process of building therapeutic alliance and initiating interventions by highlighting client strength and resilience, as well as introducing coping skills before pivoting into safety planning.

This study provides an examination of clinician experiences on the challenges of non-disclosure in an outpatient setting, where clinicians work with clients over an extended timeframe of up to 12 sessions or more, complementing studies of provider perspectives in medical settings where single patient encounters are the focus.^5^ These findings offer insights and potential strategies to address non-disclosure in military-affiliated populations where significant barriers to disclosure exist.^15–17,27^ Clinicians in this study and prior works^4^ discussed the importance of confronting their own comfort discussing suicide and using direct language during screening. Prior research has identified provider discomfort due to lack of training and experience as a barrier to screening.^5,43^ Further, the importance of therapeutic rapport in risk assessment has been noted in prior studies of provider perspectives on barriers to disclosure of STB;^4,5^ however, lack of rapport continues to be named as a barrier to disclosure.^15–17^ Together, these represent important training opportunities to facilitate provider comfort and connection during suicide risk screening. This may be especially important for providers working outside of mental health contexts where barriers to assessment may be impacted by limited time with patients and limited trainings and supports in suicide prevention. Additionally, use of standardized measures in risk assessment has been critiqued by providers and patients alike as perfunctory, repetitive, or robotic and harmful to therapeutic rapport.^4,5,16,17^ This study showcases the importance of implementing standardized screening measures within a measurement-based care framework that maintains connection and rapport.

There are several limitations to this study. This study reflects experiences of mental health clinicians working in a low to moderate acuity clinic; experiences of providers working in other settings may differ. Further, participating clinicians report high levels of experience assessing and managing suicidality, including robust training and frequent engagement with clients reporting suicidal ideation, which may impact generalizability. Additionally, this study reflects clinician interpretations of client disclosure challenges, rather than patient perspectives. However, the lived experiences and reflections of clinicians help inform specific tools and strategies that may help prevent non-disclosure among military-affiliated and civilian clients alike.

While the full extent of non-disclosure of STB among service members and veterans is unknown, both groups are at higher baseline risk for suicide yet also more prone to delay disclosure, which may further enhance risk. This research highlights the challenges clinicians face when conducting suicide risk assessment with military-affiliated clients in an outpatient mental health network as well as the strategies they employ to address non-disclosure based on their professional experiences with treating clients who delay sharing their STB. Direct communication and education around limits of confidentiality and screening/treatment goals helps clinicians address shame, stigma, and culturally specific beliefs around the risks of disclosure in military communities. These strategies align with suggestions and recommendations based on studies of patient ^16,17,25^ and provider^4,5^ perspectives. Consistent with the literature,^25,44^ training relating to how to discuss limits of confidentiality may foster therapeutic environments where clients feel comfortable disclosing STB. Further, additional research on best practices for integrating MBC into suicide risk assessment and management is needed. When implemented as a complementary aspect of care, clinicians find MBC to be a powerful tool that strengthens relationships and encourages disclosure; however, when implemented poorly or with a perfunctory approach, these same standardized measures may suppress reporting. Implementation of these strategies by clinicians serving military-affiliated clients may help to build rapport, address apprehensions, and facilitate disclosure, ultimately enabling more elevated risk clients to express and receive treatment for STB.

## Data Availability

The data used in this study is not publically available due to privacy and confidentiality requirements of the study.
